# Supplementary material for: Drug repurposing for aging research using model organisms
Source: Aging Cell. 2017 Jun 16;16(5):1006–15. doi: 10.1111/acel.12626 (PMC5595691; doi:10.1111/acel.12626)
Supplement: Supplementary file 7 — Data S1 Zip‐Archive of all report cards. [file ACEL-16-1006-s007.zip › RC_B49.pdf]

# B49

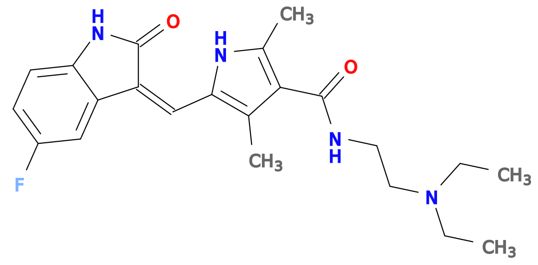


**Database identifiers**

| | |
|---|---|
| ChEMBLCompound | CHEMBL535 |
| DrugBank | DB01268 |
| CHEBI | 38940 |
| eMolecules | 23296409 |
| eMolecules | 27524016 |

## Ranking

| | Rank | Score |
|---|---|---|
| Drosophila | 104/697 | 0.787 |
| C. elegans | NA | NA |

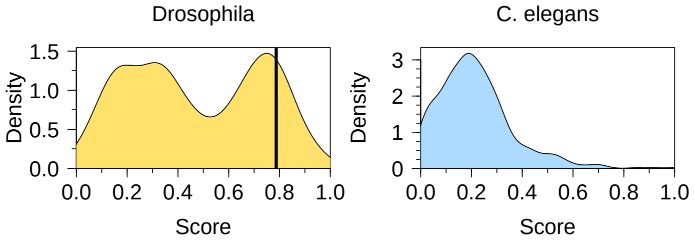


| | Ageing implication | Domain conservation | Binding site conservation | Binding affinity | Bioavailability | Lipinski | Promiscuity | Purchasability | Drug approval | **Total** |
|---|---|---|---|---|---|---|---|---|---|---|
| Drosophila | 1.0 | 0.924 | 0.779 | 0.907 | (0.9) | 0.0 | -0.0 | 0.1 | 0.1 | 0.787 |
| C. elegans | NA | NA | NA | NA | NA | NA | NA | NA | NA | NA |

## Names

- **sunitinib**
- Su-11248
- Sutent

## Roles

angiogenesis inhibitor, antineoplastic agent, EC 2.7.10.1 (receptor protein-tyrosine kinase) inhibitor, vascular endothelial growth factor receptor antagonist, immunomodulator, neuroprotective agent

## Status

| | |
|---|---|
| Approved drug (according to ChEMBL) | Yes |
| Administration Route | Oral |
| Number of Rule of 5 violations | 0 |
| Binding affinity to original target in log units (RF-Score prediction) | 7.27 |
| Burns *C. elegans* bioavailability prediction | -10.79 |

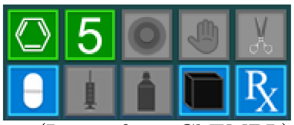

(Image from ChEMBL)

## Medical Information

**Indicatation:** For the treatment of advanced renal cell carcinoma as well as the treatment of gastrointestinal stromal tumor after disease progression on or intolerance to imatinib mesylate.
**Mechanism of action:** Sunitinib is a small molecule that inhibits multiple RTKs, some of which are implicated in tumor growth, pathologic angiogenesis, and metastatic progression of cancer. Sunitinib was evaluated for its inhibitory activity against a variety of kinases (>80 kinases) and was identified as an inhibitor of platelet-derived growth factor receptors (PDGFRa and PDGFRb), vascular endothelial growth factor receptors (VEGFR1, VEGFR2 and VEGFR3), stem cell factor receptor (KIT), Fms-like tyrosine kinase-3 (FLT3), colony stimulating factor receptor Type 1 (CSF-1R), and the glial cell-line derived neurotrophic factor receptor (RET). Sunitinib inhibition of the activity of these RTKs has been demonstrated in biochemical and cellular assays, and inhibition of function has been demonstrated in cell proliferation assays. The primary metabolite exhibits similar potency compared to sunitinib in biochemical and cellular assays.
**Toxicity:** The maximally tolerated dose for rat, mouse, and dog when given orally is greater than 500 mg/kg. The maximally tolerated dose of a non-human primate is greater 1200 mg/kg.
**Metabolism:** Sunitinib is metabolized primarily by the cytochrome P450 enzyme, CYP3A4, to produce its primary active metabolite, which is further metabolized by CYP3A4.
**Retail:** Pfizer Inc.
Sutent 12.5 mg capsule - 93.64 USD / capsule
Sutent 25 mg capsule - 187.28 USD / capsule
Sutent 50 mg capsule - 333.39 USD / capsule

(Information from DrugBank)

## Compound Target Characteristics

### Tyrosine-protein kinase ITK/TSK

Best gene implication in ageing for this target family came from gene P08630 annotated in UniProt release 2014_02. Annotation GO 8340 (determination of adult lifespan) was Inferred from Mutant Phenotype

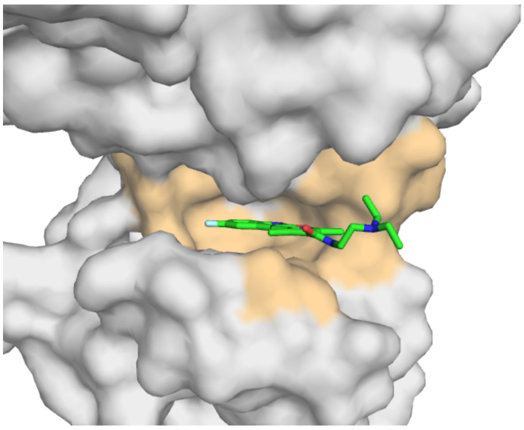

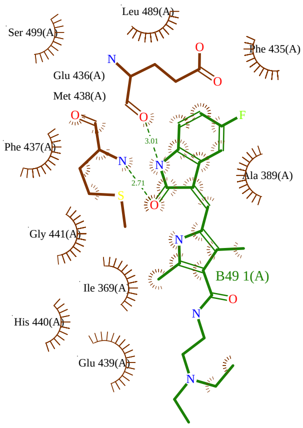

| protein | amino acids contacts (binding site) |
|---|---|
| PDB:3miy:chainA:Q08881 | I A K F E F M E H G L S D |
| sp:Q08881:ITK_HUMAN | I A K F E F M E H G L S D |
| tr:D4A7W7:D4A7W7_RAT | I A K F E F M E H G L S D |
| tr:Q8CAW3:Q8CAW3_MOUSE | I A K F E F M E H G L S D |
| tr:A1A560:A1A560_MOUSE | I A K F E F M E H G L S D |
| tr:Q5STT8:Q5STT8_MOUSE | I A K F E F M E H G L S D |
| sp:Q03526:ITK_MOUSE | I A K F E F M E H G L S D |
| sp:P08630:BTKL_DROME | L A K T E Y M K H G L A D |

| | whole protein | | domain-based | | contact-based | |
|---|---|---|---|---|---|---|
| protein | ident | simil | ident | simil | ident | simil |
| PDB:3miy:chainA:Q08881 | 0.99 | 0.99 | 1.0 | 1.0 | 1.0 | 1.0 |
| sp:Q08881:ITK_HUMAN | 1.0 | 1.0 | 1.0 | 1.0 | 1.0 | 1.0 |
| tr:D4A7W7:D4A7W7_RAT | 0.94 | 0.97 | 0.97 | 0.99 | 1.0 | 1.0 |
| tr:Q8CAW3:Q8CAW3_MOUSE | 0.53 | 0.55 | 0.97 | 0.99 | 1.0 | 1.0 |
| tr:A1A560:A1A560_MOUSE | 0.94 | 0.98 | 0.97 | 0.99 | 1.0 | 1.0 |
| tr:Q5STT8:Q5STT8_MOUSE | 0.94 | 0.98 | 0.97 | 0.99 | 1.0 | 1.0 |
| sp:Q03526:ITK_MOUSE | 0.93 | 0.97 | 0.97 | 0.99 | 1.0 | 1.0 |
| sp:P08630:BTKL_DROME | 0.35 | 0.62 | 0.55 | 0.85 | 0.62 | 0.78 |

### Btk29A (FBgn0003502) associated phenotypes

cell shape defective, lethal - all die before end of first instar larval stage, mating defective, partially, short lived, size defective, some die during first instar larval stage

(Information from FlyBase)

### Btk29A (UniProt:P08630) annotation

**Function**: Required for proper ring canal development. Also required for the development of male genitalia and for adult survival. (PubMed:10330180, PubMed:9655810).
**Cofactor**: Zn(2+)Note=Binds 1 zinc ion per subunit. ;
**Subcellular location**: Note=Ring canals.
**Tissue specificity**: Ring canals in the egg chambers and imaginal disks of third-instar larvae. (PubMed:10330180, PubMed:3110602, PubMed:9655810).
**Developmental stage**: Expressed both maternally and zygotically. Predominantly in early to middle embryogenesis, in larvae and adult females. (PubMed:10330180, PubMed:3110602).
**Disruption phenotype**: Flies exhibit shortened copulatory duration (due to incomplete fusion of the left and right halves of the apodeme that holds the penis during copulation) and reduced adult-stage life span. (PubMed:10330180).

(Information from UniProt)
